# Editorial: Social psychological process and effects on the law

**DOI:** 10.3389/fpsyg.2022.990167

**Published:** 2022-08-16

**Authors:** Colleen M. Berryessa, Clare S. Allely, Melissa de Vel-Palumbo, Yael Granot

**Affiliations:** ^1^School of Criminal Justice, Rutgers University, Newark, NJ, United States; ^2^School of Health and Society, University of Salford, Manchester, United Kingdom; ^3^College of Business, Government and Law, Flinders University, Adelaide, SA, Australia; ^4^Department of Psychology, Smith College, Northampton, MA, United States

**Keywords:** social psychology, law, human behavior, criminal justice, decision-making

Studying social psychological processes entails disentangling how people perceive, interact in, and react to our social world. This framework has been increasingly applied to studying law, with growing interest in the ways in which social contexts intersect with legal institutions and decision-making. Although the law may be viewed as insulated from social contexts, it is in fact ever-changing and shaped over time by society. Work at this intersection offers insights into how social psychology can impact the law, but also informs the law about the ways in which the public engages with and perceives legal principles, practices, and proceedings.

Although research at this intersection has begun to grow in recent years, many areas of empirical and theoretical work in this area continue to be under-studied, particularly across different countries and legal systems, those using more interdisciplinary frameworks in the study of these relationships, and in considering broader understandings and applications of social psychological processes to studying the law.

The 13 papers included in this Research Topic approach varied aspects of social cognition and its relationship to legal processes, providing important guidance on how we might explore these questions in future international work across different jurisdictions and countries. We are thrilled that researchers represented in this international collection hail from Australia, Canada, Chile, China, Hong Kong, Italy, Japan, the Netherlands, Spain, Sweden, and the United States.

Grosfeld et al. survey members of the European Union (EU), finding that value alignment, particularly in relation to binding values, plays a significant role in affecting the public's views on the perceived legitimacy of the Court of Justice of the EU (CJEU) and the EU more broadly.

Younan and Martire present two experimental studies to U.S. participants on the effects of expert likeability. They find that likeability may influence judgments on experts' persuasiveness and testimony quality, but may not necessarily affect support for particular sentencing outcomes.

Kurinec and Weaver also use two online experiments to show that the speech stereotypicality of Black Americans may activate racial stereotypes and racial phenotype bias, which influence suspect descriptions and eyewitness identifications.

Albrecht and Nadler test how the composition of crime news articles contributes to reader perceptions of moral blameworthiness and corresponding punishment attributions of vehicular homicide offenders. This study suggests that lay support for more severe punishment is affected by participants' characteristics, particularly political affiliation, when the immigration status of a suspect is provided and uncovers how differential reporting on suspects' personal characteristics may affect public views on blameworthiness.

Shang et al. present a study that measures types of social behavior, such as empathy, perspective-taking, and self-control, in Chinese adolescents in order to further work on whether age should continue to be the primary attribute by which to judge a juvenile's criminal responsibility. Their findings support the notion that legal systems may want to consider juvenile responsibility in terms of the social and interpersonal maturity and decision-making, rather than solely in terms of age.

Pettersson et al. study Swedish police officers and their ability to conduct investigative interviews with intoxicated witnesses, primarily looking at how police decision-making and perceptions of witness credibility may be biased by pre-existing social norms. This study adds to the existing literature in the field by showing that breath alcohol concentration far lower than the legal maximum still significantly affected officers' views on witness credibility.

Watamura et al. from Japan, develop a ratio measure to see how people weigh and justify different punishment philosophies when considering sentences for child abuse cases. Results show that ratio justifications differ across cases involving either severe or moderate abuse, with both retribution and utilitarian justification considered in the sentencing decisions of such cases.

Ansems et al. study Dutch criminal court hearings involving defendants with non-Western backgrounds to examine how prior discrimination and outcome judgments might interfere with the effects of procedural justice. Their findings help to illuminate the importance of promoting procedural justice in Dutch courts as a way to decrease social costs associated with continued justice system involvement.

Guan and Lo present a systematic review on drug offending within a certainty–severity framework of punishment, covering a wide body of literature on the importance of exposing certain types of information on punishment as a way to deter drug offending. Main themes identified in this literature focus on restrictive deterrence strategies, particularly surrounding pre-arrest, deterrability, and perceptions of risk, which suggest expanding future work on after-arrest strategies and across different types of drug offenders.

Ewanation and Maeder use an online experiment of U.S. participants to study the effects of a defendant's race and the presence or absence of expert testimony on jurors' perceptions of recanted confessions. Results support a “watchdog hypothesis” as White mock-jurors were found to be more receptive to legally relevant evidence when a defendant was identified as Black.

Angioletti et al. using common moral dilemmas from psychology research, demonstrate how individual, situational, contextual, and internal factors may influence the moral decision-making of lawyers in Italy. Results show that lawyers' internal states (e.g., interoceptive ability) may influence their fairness in decision-making during trial.

Saad et al. in an experimental study of New Jersey parole officers, find that officers' implicit social cognition may influence their behaviors toward and empathy for those whom they supervise. Findings may help to improve therapeutic and supervision relationships between officers and their clients.

Camplá et al. assess informal reasoning and biases that may affect the decision-making of Chilean legal actors in rape cases. Results find that these actors commonly overestimate probabilities of false or unfounded allegations and myths about sexual offending, and show attributional biases toward victims.

Ultimately, this paper collection represents an expansive and comprehensive account of international research on widespread ways in which law and social psychology interact. These issues are not only important to common legal practices, such as eyewitness identification, interviewing, or trial proceedings, and how they may be influenced by discrimination, bias, and other social processes, but also when considering how social psychological processes could influence larger philosophical questions on why we punish, why we use and support various legal practices, and the design and evaluation of legal rules. Thus, interactive relationships between law and social psychology should be viewed as “two-way streets” that will continue to shape criminal-legal outcomes across the globe moving forward.

## Author contributions

All authors recognize CB leadership and substantial and direct contribution to this publication. All authors intellectually contributed to this work and approved it for publication.

## Conflict of interest

The authors declare that the research was conducted in the absence of any commercial or financial relationships that could be construed as a potential conflict of interest.

## Publisher's note

All claims expressed in this article are solely those of the authors and do not necessarily represent those of their affiliated organizations, or those of the publisher, the editors and the reviewers. Any product that may be evaluated in this article, or claim that may be made by its manufacturer, is not guaranteed or endorsed by the publisher.

